# Recruitment challenges in surgical trials: lessons from the crisp trial

**DOI:** 10.1186/1745-6215-14-S1-P27

**Published:** 2013-11-29

**Authors:** K Pike, GD Angelini, BC Reeves, DP Taggart, CA Rogers

**Affiliations:** 1 University of Bristol, Bristol, UK; 2 University of Oxford, Oxford, UK

## Background

The results of surgical trials are often challenged because of concerns about the experience and skill of the surgeons taking part. When well-established alternative surgical procedures exist, individual surgeons are usually more proficient in, and favour, a single approach. Conventional within-surgeon randomisation requires surgeons to be proficient in both procedures, which may skew the surgical expertise towards one technique. Expertise-based randomisation overcomes this limitation. We describe our experience of implementing expertise-based randomisation in the context of the CRISP trial to compare on-pump and off-pump CABG in high-risk patients.

## Methods

Non-emergency patients with a EuroSCORE>=5 were eligible to participate. Elective patients were approached and consented at the pre-assessment clinic. Information was faxed to urgent patients waiting in nearby “feeder” hospitals and they were consented on transfer to the specialist centre. Following randomisation surgery was arranged with the appropriate expert surgeon.

## Results

The trial struggled to recruit. Barriers to recruitment included (a) patients unwilling to be randomised to a surgeon they had not met; (b) surgeons unwilling to “share” patients; (c) unavailability of expert surgeon to operate within NHS target time; (d) insufficient time to arrange surgery. After 18 months the trial was closed prematurely. 106 patients had been recruited from a study target of 5200 randomised patients.

## Conclusion

Expertise-based randomisation may not be possible in a tertiary referral setting in the UK and may be more easily achieved overseas where healthcare is organised differently. Including integrated qualitative research to gain full understanding of trial issues may have increased patient participation.

